# Integrated emergy and economic evaluation of the dominant organic rice production systems in Jiangsu province, China

**DOI:** 10.3389/fpls.2023.1107880

**Published:** 2023-03-24

**Authors:** Pinglei Gao, Haoyu Wang, Guojun Sun, Qiang Xu, Zhi Dou, Erjia Dong, Wenge Wu, Qigen Dai

**Affiliations:** ^1^ Jiangsu Key Laboratory of Crop Genetics and Physiology, Jiangsu Key Laboratory of Crop Cultivation and Physiology, Jiangsu Co-Innovation Center for Modern Production Technology of Grain Crops, Research Institute of Rice Industrial Engineering Technology, Yangzhou University, Yangzhou, China; ^2^ Agricultural and Rural Bureau of Jintan District, Changzhou, China; ^3^ Anhui Pujiwei Modern Agriculture Group Co., Ltd., Tongling, China; ^4^ Rice Research Institute, Anhui Academy of Agricultural Sciences, Hefei, China

**Keywords:** rice-green manure rotation, integrated planting-breeding, rice-duck co-culture, rice-crayfish co-culture, ecological economic benefits, environmental sustainability

## Abstract

Changing from conventional to organic farming might have fewer negative environmental impacts because of the avoidance of synthetic fertilizer and chemical pesticides. In this study, the economic viability and environmental and sustainability performance of the four dominant organic (rice-green manure rotation (RG), rice-duck co-culture (RD), rice-crayfish co-culture (RCF) and rice monoculture (RM)) and one conventional (rice monoculture (CRM)) rice production modes were evaluated in Jiangsu Province, China. Compared with the CRM mode, organic rice production increased economic benefits density and improved the economic benefit of crop land and irrigation water use. With the lowest total emergy input and the highest rice yield, the CRM mode showed the highest ecological efficiency in converting resources to total available energy content and nutrition density unit among the five rice production modes. However, the RCM mode showed higher environmental pressure and lower sustainability than the four organic modes due to the larger proportion of nonrenewable emergy input. The RM mode was the most uneconomic organic rice production mode with the highest cost input and the lowest product output but had relatively higher sustainability due to the higher proportion of renewable resources to total emergy inputs. Compared with the RM mode, the value-to-cost ratio, economic benefit density and benefit-cost ratio were increased in the RG, RD and RCF modes. Although the RD and RCF modes had higher efficiency in converting resources to total nutrition density units and monetary value, they imposed higher environmental pressure with a lower renewable fraction and emergy sustainability index than those in the RM mode. The RG mode had higher emergy utilization efficiency and the highest renewable fraction and emergy sustainability index among the four organic rice production modes. Considering the ecological and economic effects, the RG mode was conducive to improving the economic viability and sustainability of organic rice production.

## 1 Introduction

Rice (*Oryza sativa* L.) is a major part of the daily diet of about 3 billion people and an important part of global food security, with a worldwide planting area of 163 million ha (Frei and Becker, 2005; Woolston, 2014). In the past several decades, with the application of chemical fertilizers and pesticides, rice yield has increased greatly. However, the long-term and excessive application of these agrochemicals raises environmental concerns such as soil pollution and degradation, freshwater eutrophication, increased pesticide resistance and pesticide residues in the grain (Sutton et al., 2013; Tilman et al., 2002). Generally, organic agriculture is considered to have fewer negative environmental impacts because of the avoidance of synthetic fertilizer and chemical pesticides (Nemecek et al., 2011; Meng et al., 2017; He et al., 2018).

Changing from conventional farming to organic farming may cause concerns about fertilizer application and pest control because of the avoidance of synthetic agrochemicals. Applying green manure to paddy soil is considered to be a good management practice instead of the use of synthetic fertilizers, which can improve soil sustainability by reducing soil erosion and ameliorating soil physical properties (Lou et al., 2011), and increasing soil organic matter and fertility (Thorup-Kristensen et al., 2003), and nutrient retention (Drinkwater et al., 1998; Dennis et al., 2010). The application of green manure was also reported to reduce the occurrence of insect pests and plant diseases (Naz et al., 2015). More than 90% of world’s paddy fields are under shallow water, which could provide a suitable environment for a wide range of aquatic animals and waterfowl, including fish, crab, crayfish, soft-shell turtles and ducks (Pirdashti et al., 2015; Zhang et al., 2016; Xu et al., 2021). Coculture of rice and waterfowl or aquatic animals has been proposed as a strategy to reduce the dependence on external material and energy inputs, such as pesticides, fertilizers and forage, by efficient internal recycling (Cavalett et al., 2006) while the risks of environmental pollution related to rice production (Xie et al., 2011; Xu et al., 2021) is also decreasing. Therefore, rice-green manure rotation and rice-integrated planting-breeding systems are widely adopted in organic rice planting.

Jiangsu province is a major rice producer and consumer in China, with a perennial rice planting area of approximately 2.2 million ha and a total output of 18 million t, accounting for 7% and 10% of the national rice planting area and production, respectively (National Bureau of Statistics, 2020). In recent years, with the improvement of people’s living standards and environmental awareness, the demand for organic rice has increased with a concomitant increment in the planting area of organic rice. According to the Jiangsu Provincial Department of Agriculture and Rural Development in 2020, the total planting area of organic rice in Jiangsu Province is 4410 ha, distributed among a variety of organic production modes that include rice-green manure rotation (RG), rice-duck co-culture (RD), rice-crayfish co-culture (RCF), rice monoculture (RM), rice-crab co-culture and rice-frog co-culture. RG, RD, RCF and RM are the four dominant organic rice production modes, accounting for 45%, 24%, 16% and 5% of the organic rice planting area in Jiangsu Province, respectively. What are the ecological-economic characteristics of these dominant modes for organic rice production? What is the optimal sustainable mode for organic rice production that can balance environmental and economic benefits? Regarding the applicability of these currently dominant models, what are the main limiting factors affecting the optimal sustainable mode? Evidently, all of these questions need to be answered by further exploring eco-economic theory to guide the formulation of future conservation and production strategies.

It is necessary to consider both economic and ecological problems in the pursuit of sustainable development, which is obviously a problem beyond the ability of pure economic or environmental analysis (Lu et al., 2017). Emergy accounting (EMA) is a method of comprehensive accounting for environmental and economic systems according to the concept of emergy proposed by the American ecologist Howard Odum (Odum, 1986; Odum, 1996). In emergy synthesis, based on equivalent unit (solar emjoules or sej), the flows of energy and matter can be described by multiplying the corresponding transformations (Jafari et al., 2018). Therefore, emergy analysis has been put forward as the bridge between economy and ecology (Ulgiati and Brown, 1998; Lan et al., 2002), which has also been proven to be particularly suitable for evaluating systems with an interface between the “human” and “nature” systems, such as agro-ecosystems (Brown and Ulgiati, 1997; Ulgiati and Brown, 1998; Lu et al., 2010; Zhang et al., 2011; Zhang et al., 2013; Lu et al., 2017). In the past two decades, combination of emergy analysis with various indices and ratios has been been widely applied to evaluate the sustainability of agricultural production systems at different types and scales. Xi and Qin (2009) used emergy assessment to evaluate the economic benefits and sustainability of rice-duck coculture and wheat–rice rotation systems and demonstrated that rice-duck coculture increased emergy efficiency and decreased environmental pressure compared with wheat–rice rotation. A comparative study on emergy and economic evaluation of three lotus root production systems in reclaimed wetlands (Lu et al., 2017) indicated that compared to the pure lotus root production mode, the lotus-fish production mode was more sustainable and had higher economic viability, while the lotus-shrimp production mode did not improve the ecological-economic characteristics of lotus culture as expected. Xu et al. (2019) compared the eco-economic performance of rice monoculture, conventional, and optimized rice-crab production modes in northeastern China and suggested that an optimized rice-crab system had better economic and ecological effects than the rice monoculture and traditional rice-crab systems. Hou et al. (2021) conducted nutrient use efficiency, economic, and emergy analyses of rice monoculture, rice-crayfish rotation, and rice-crayfish coculture systems and suggested that rice-crayfish systems had better economic benefits, better fertilizer nutrient use efficiency, and lower environmental pressure but decreased the renewable fraction and emergy yield ratio and sustainability index compared with rice monoculture; the authors concluded that rice-crayfish systems were not a panacea for sustaining cleaner food production. Most of these studies were based on conventional rice production and focused on comparisons with conventional agricultural production systems. However, there are significant differences between organic and conventional rice production in terms of cultivation mode, fertilizer application, pest management, rice yield and price. These differences in inputs and outputs will inevitably lead to differences in emergy and economic evaluation between organic and conventional rice production systems. Therefore, it is of great significance to study the ecological-economic characteristics of different organic rice production modes to explore the balance of economic and environmental benefits and provide decision-making support for organic rice production.

In the current study, the integration of economic and emergy methods was used to evaluate and compare the economic viability and environmental and sustainability performance of the four dominant organic (RG, RD, RCF and RM) and one conventional (conventional) rice production modes in Jiangsu Province. The optimal sustainable system for organic rice production that could balance environmental and economic benefits is suggested, and the main limiting factors are briefly discussed.

## 2 Materials and methods

### 2.1 Study sites and system description

**Figure 1:**
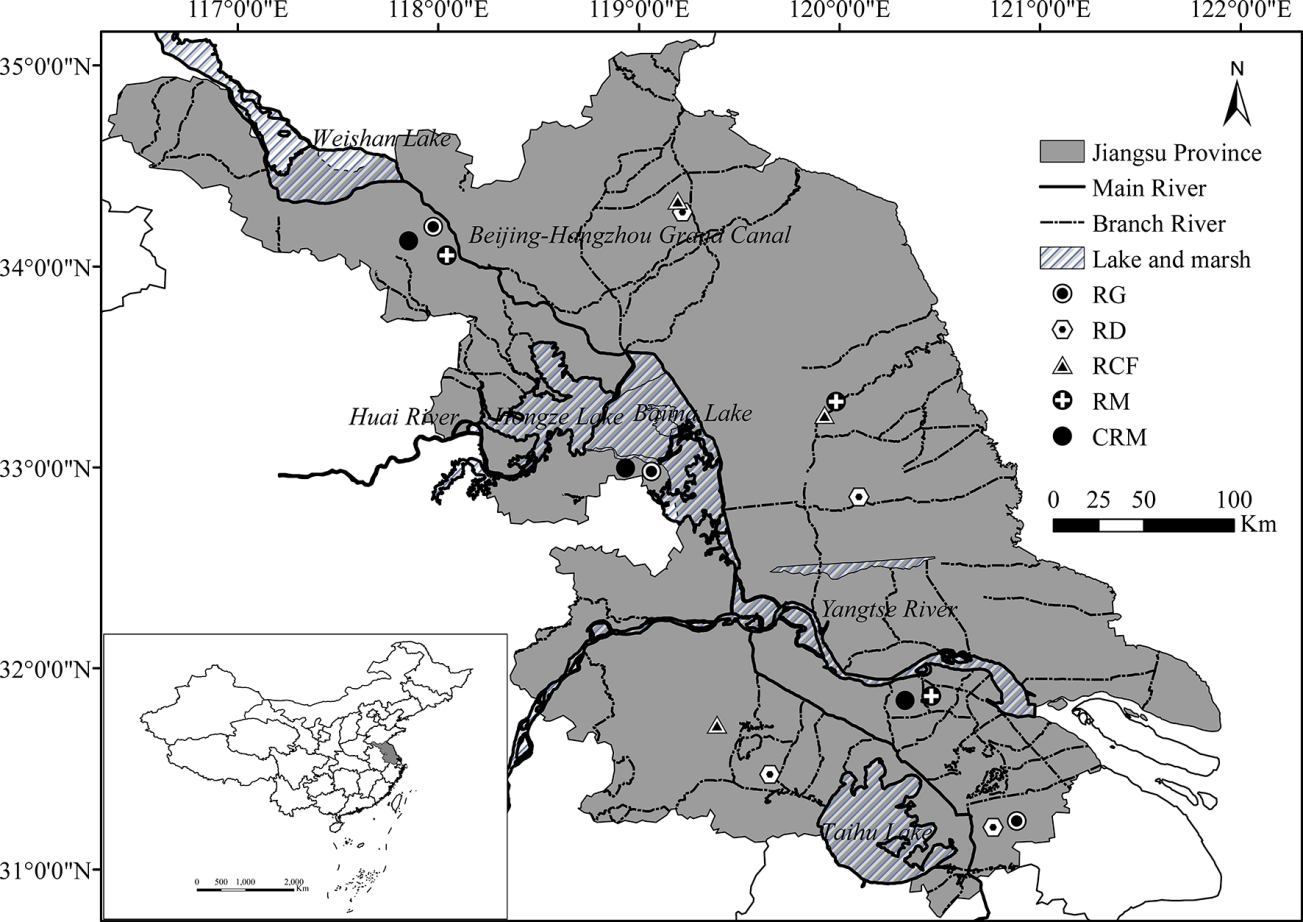
Survey sites of the five rice production modes. RG, Rice-green manure rotation mode for organic rice production; RD, rice-duck coculture mode for organic rice production; RCF, rice-crayfish coculture mode for organic rice production; RM, rice monoculture mode for organic rice production; CRM, rice monoculture mode for conventional rice production.

Jiangsu Province is located in the center of the east coast area of mainland China (30°45′-35°20′N, 116°18′-121°57′E, **Figure 1**). Jiangsu is dominated by plains (87%), with a land area of 103229.17 km^2^. Jiangsu has a crisscrossed dense water network, which runs from north to south by the Beijing-Hangzhou Great Canal, straddles the Yangtze River and the Huaihe River, and contains a number of medium and large lakes. Jiangsu belongs to the East Asian monsoon climate zone, which is located in the climate transition zone between the subtropical and warm temperate zones, with an annual precipitation of 704-1250 mm and an annual average temperature between 13.6-16.1 °C. Affected by the monsoon, the north–south temperature difference is obvious, and the precipitation is greater in the south than in the north and greater along the coast than inland.

The unique landform, hydrology and climate environment of Jiangsu make the whole territory suitable for rice planting. Rice is the first and foremost grain crop in Jiangsu Province, and its perennial planting area and yield account for approximately 42% and 55% of the total planting area and yield of grain crops in the province, respectively (National Bureau of Statistics, 2020). A variety of organic rice production modes are also widely distributed in all cities of Jiangsu Province. RG, RD, RCF and RM are the four dominant organic rice production modes and CRM is the most basic conventional rice production mode, which are the subject of this research. Although the production process of each rice production mode is relatively consistent, differences in geographical environment and agronomic operation among regions lead to differences in the input and output of each rice production mode. Therefore, to ensure the objectivity of the results, the surveys were conducted at three different sites in Jiangsu Province for each rice production mode (**Figure 1**). The specific production programs and field areas of the five rice production modes are presented in **Table 1**.

**Table 1:** Characteristics and production processes of the five rice production modes.

Item^a^	RG	RD	RCF	RM	CRM
**Area**	1968 ha	1069 ha	707 ha	164 ha	86 ha
**Rice planting^b^**	8.33E+05 seedlings/ha	7.69E+05 seedlings/ha	6.92E+05 seedlings/ha	8.33E+05 seedlings/ha	8.33E+05 seedlings/ha
**Rice harvest^c^**	7275 kg/ha	7250 kg/ha	7100 kg/ha	6675 kg/ha	9750 kg/ha
**Green manure^d^**	45 kg/ha seeds				
**Input of duck or crayfish^e^**		13.98 kg/ha ducklings	450 kg/ha juvenile crayfish		
**Harvest of duck or crayfish^f^**		382.5kg/ha	1365 kg/ha		
**Length of cropping cycle**	330 d	150 d	180 d	150 d	

^a^RG: Rice-green manure rotation mode for organic rice production; RD: rice-duck coculture mode for organic rice production; RCF: rice-crayfish coculture mode for organic rice production; RM: rice monoculture mode for organic rice production; CRM: rice monoculture mode for conventional rice production. ^b^Rice planting was conducted in early July in each rice production mode. ^c^Rice harvest was conducted in late October in each rice production mode. ^d^Chinese Milk Vetch (Astragalus sinicus), sowing in early November and ploughing into the soil in late May next year. ^e^The input of duck was in mid-June and the input of crayfish was in mid-April (300 kg/ha) and mid-June (150 kg/ha). ^f^The harvest of duck was in late August and that of crayfish was in late July and late September.

### 2.2 Economic analysis

In order to evaluated the economic viability of the four organic rice production modes, a set of economic indicators including the value-to-cost ratio (VCR), benefit-cost ratio (BCR) and economic benefits density (EBD), (**Table 2**), were calculated and integrated with the results of emergy evaluation to allow for a complete exploration of the ecological-economic characteristics of the four modes. The data of price and quantity and materials were provided by local farmers and agricultural product distributors through formal survey questionnaires.

**Table 2:** Economic and emergy indices employed in this study and their expressions.

Item	Expression	Source
Economic index		
Value to cost ratio (VCR)	VCR = MY/MI	Xu et al., 2019
Economic benefifits density (EBD)	EBD (¥/ha/yr) = (MY - MI)/area	Xu et al., 2019
Benefifit to cost ratio (BCR)	BCR (%) = EBD/MI × 100	Xu et al., 2019
Irrigation water based on unit benefit (IW_¥_)	IW_¥_ (m^3^/¥) = irrigation water/(MY−MI)	Hou et al., 2021
Land occupation based on unit benefit (LO_¥_)	LO_¥_ (m^2^/¥) = area/(MY−MI)	Hou et al., 2021
Emergy index		
Unit Emergy Value (UEV)	UEV = U/Y	Odum, 1996
Renewable fraction (%R)	R = 100 × (L_R_ + F_R_)/U	Odum, 1996
Emergy Yield Ratio (EYR)	EYR = U/F	Brown and Ulgiati, 2004
Environmental Loading Ratio (ELR)	ELR = (L_N_ + F_N_)/(L_R_ + F_R_)	Ulgiati et al., 1994
Emergy Sustainability Index (ESI)	ESI = EYR/ELR	Brown and Ulgiati, 1997

MI, total cost; MY, total market value of output; U, Total emergy inputs; Y, Yield, i.e. the product generated by a process.

### 2.3 Emergy evaluation

**Figure 2:**
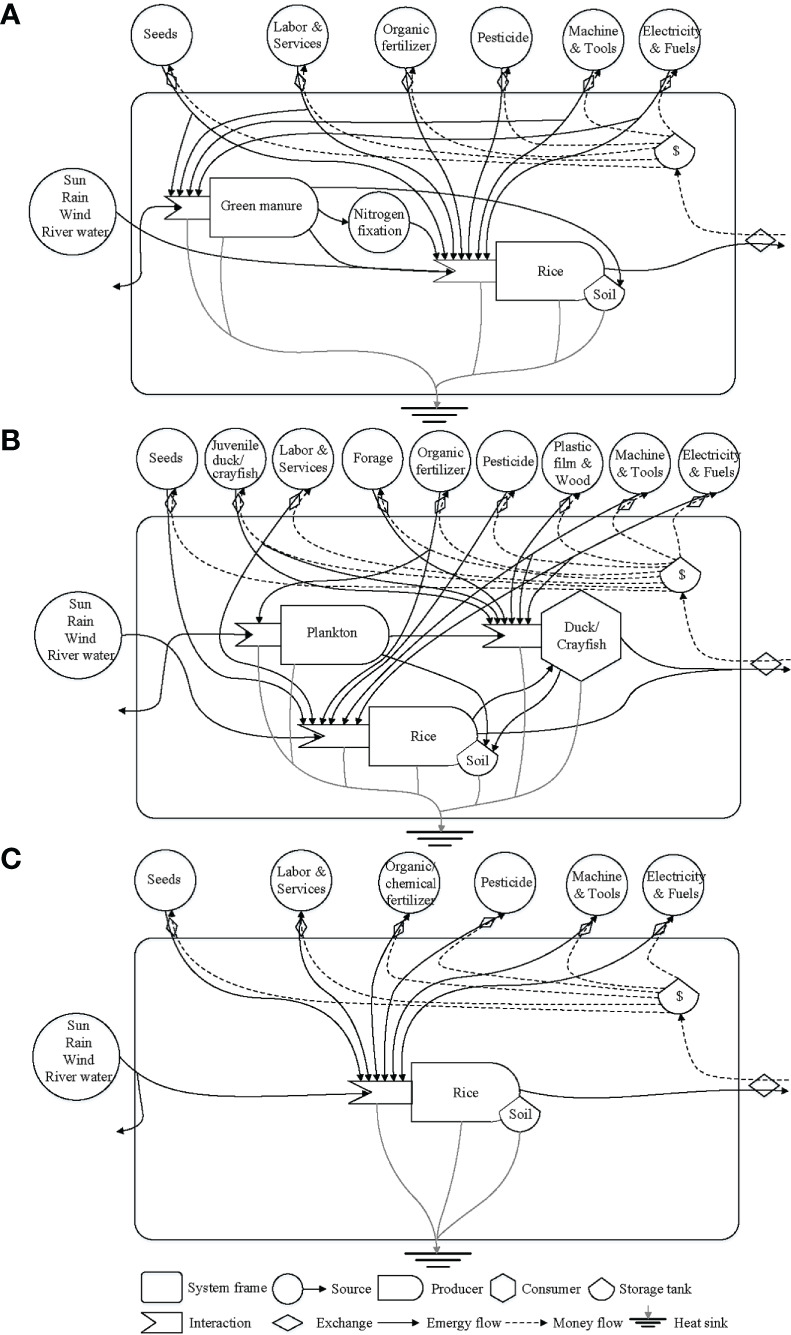
Aggregated energy flow diagrams of rice-green manure rotation **(A)**, rice-duck/crayfish co-culture **(B)** and organic/conventional rice monoculture **(C)**.

The emergy analysis process is based on the work of Odum (1986); energy flow diagrams and emergy sources driving the different rice production systems are shown in **Figure 2**. In this study, the inputs and outputs of material, monetary and energy flows among different rice production modes were averaged (data per site) and converted into emergy units based on the planetary baseline of 12.0E+24 sej/year (Brown et al., 2016), and placed in emergy synthesis tables (**Supplementary Material, Tables A, B, C, D, E**), where all emergy sources were further categorized into three types: 1) free local renewable resources (L_R_), 2) free local nonrenewable resources (L_N_), and 3) economic imported resources (F) according to the definition by Xu et al. (2019). The economic imported resources (F) were also divided into renewable (F_R_) and nonrenewable (F_N_) imported flows based on their renewability fractions (RNFs) (Odum, 1986; Brown and Ulgiati, 1997). Green manure, nitrogen fixation, straw, and feces of duck and crayfish were reused in each system; therefore, they were classified as feedback yield energy, and based on emergy theory by Brown and Ulgiati (1997), their emergy values were zero. In addition to the analysis of the the input and output compositions, the environmental efficiency and sustainability of the five rice production modes were evaluated by several emergy indices (**Table 2**) including their unit emergy value (UEV), renewable fraction (%R), environmental loading ratio (ELR), emergy yield ratio (EYR), and emergy sustainability index (ESI) (Odum, 2000). In order to figure out the proportion of the total emergy usage derived from the information contained in the labor and background infrastructure, the UEVs of rice, duck and crayfish (namely transformity) were calculated with or without labor and service (L&S), and the specific emergy (namely specific emergy) of products was also weighed in the evaluation of production efficiency per unit weight of each system.

### 2.4 Data source and processing

The inputs and outputs surveys of the five rice production systems (RG, RD, RCF, RM and CRM) were conducted through survey questionnaires in the north, middle and south of Jiangsu Province. The survey of each organic rice production system was carried out at three different sites (**Figure 1**), and there were 84 representative questionnaires from 8 cities. Meteorological data, such as the solar radiation, precipitation and wind speed of each survey site, were obtained from the China Meteorological Data Service Centre (http://data.cma.cn). The details of inputs included the amounts of organic/chemical fertilizer, pesticide, electricity, machine and tools, diesel, services, labor, lime, duck shelter (wood), duck and crayfish fence (wood sticks and plastic film), rice and green manure seeds, juvenile duck and crayfish and forage. The details of the outputs included the yield of rice, green manure, duck, crayfish and straw and duck and crayfish feces. All machines involved in each system were converted into annual flows according to their expected lifespan (working hours) of the machinery related, which was valued to be 18000, 18000, 4000, 1200 and 5000 hours for the tractor, excavator, pump, rice transplanter and combine harvester, respectively. All these items were converted to annual flow (i.e., nutrient, economic, and emergy) per hectare, and all outliers were eliminate from calculation. Details on the calculation procedure of raw data are shown in the Supplementary Materials.

To compare the ecological-economic characteristics in each rice production system, one-way analyses of variance (using a least significant difference test at a significance level of 5%) were conducted to determine the difference in emergy flows, transformatives and specific emergies and emergy and economic (including total cost and output) indices (data per site) among the different rice production systems. All data were tested for homogeneity of variance (Levene’s test, P > 0.05) and normality (Shapiro–Wilk test, P > 0.05) before being subjected to ANOVA. All of the above analyses were conducted using SPSS v. 20.0 (SPSS Inc., Chicago, IL, USA) and Origin 8.0 (Origin Lab, Hampton, MA, USA) was used to generate the graphs.

## 3 Results

### 3.1 Economic analysis

A comparison of the economic indices of the five rice production modes is presented in **Table 3**. Total cost (MI) and market value of output (MY) differed (P < 0.001, P < 0.001) among the five modes. Among the five modes, MI of the CRM mode was the lowest and that of the RM mode was the highest and those of the other three mode were similar. The major costs were labor (16.02% of MI), chemical fertilizer (15.15% of MI) and tillage (14.92% of MI) in the CRM mode, while labor, organic fertilizer and tillage were the major costs of the RM mode, which accounted for 52.81%, 23.27% and 5.87% of the MI, respectively. Although 52.81% of organic fertilizer was decreased in the RG mode compared with the RM mode, labor, organic fertilizer and tillage were still the major costs of the RG mode, which accounted for 56.77%, 11.74% and 6.48% of the MI, respectively. Compared with the RM mode, 53.55% and 58.33% of organic fertilizer and 50.03% and 44.58% of labor were decreased in the RD and RCF modes, respectively. The major costs were labor (29.23% of MI), forage (16.58% of MI) and organic fertilizer (11.97% of MI) in the RD mode and labor (31.69% of MI), juvenile crayfish (15.15% of MI) and forage (10.91% of MI) in the RCF mode. Although the rice yield of was the highest, the total market value of output (MY) in the CRM mode was the lowest among the five rice production modes due to the low price of conventional rice. The MY of the RM was the lowest among the organic rice production modes because of the lowest rice yield. The MY of the RCF mode was the highest among the five modes. The MY of RCF, RD and RG was 69.42%, 57.53% and 8.99% higher than that of the RM mode, respectively. The crayfish yield accounted for 37.19% of the MY in the RCF mode and 31.04% of the MY in the RD mode was attributed to duck yield.

**Table 3:** Comparison of economic indices of the five rice production modes.

Item^a^	RG	RD	RCF	RM	CRM
MI (1000 ¥/ha)	26.62 ± 0.30B	26.16 ± 0.21B	26.60 ± 0.03B	28.97 ± 0.31A	11.39 ± 0.07C
MY (1000 ¥/ha)	59.66 ± 0.94C	86.23 ± 0.89B	92.73 ± 1.49A	54.74 ± 0.36D	27.30 ± 0.36E
VCR (%)	2.24 ± 0.04D	3.30 ± 0.06B	3.49 ± 0.06A	1.89 ± 0.01E	2.40 ± 0.03C
EBD (1000 ¥/ha/yr)	33.04 ± 0.92C	60.06 ± 1.08B	66.13 ± 1.51A	25.77 ± 0.16D	15.91 ± 0.02E
BCR (%)	124.16 ± 3.84D	229.66 ± 5.91B	248.63 ± 5.92A	88.97 ± 1.10E	139.74 ± 3.35C
IW_¥_ (m^3^/¥)	0.17 ± 0.01C	0.10 ± 0.00E	0.12 ± 0.00D	0.22 ± 0.00B	0.35 ± 0.01A
LO_¥_ (m^3^/¥)	0.30 ± 0.01C	0.17 ± 0.00D	0.15 ± 0.00D	0.39 ± 0.00B	0.63 ± 0.01A

a RG, Rice-green manure rotation mode for organic rice production; RD, rice-duck coculture mode for organic rice production; RCF, rice-crayfish coculture mode for organic rice production; RM, rice monoculture mode for organic rice production; CRM, rice monoculture mode for conventional rice production. MI, total cost; MY, total market value of output; VCR, value to cost ratio; EBD, economic benefits density; BCR, benefit to cost ratio; IW_¥_, irrigation water based on unit benefit; LO_¥_, land occupation based on unit benefit. The price of organic rice, conventional rice and crayfish was 8.2, 2.8 and 25 ¥/kg, respectively, and the duck price was 105 ¥/duck (average weight of each duck was 1.5 kg) based on the survey in 2020. Values followed by different uppercase letters within a row are significantly different according to the LSD test at P < 0.05.

There were significant differences in the value-to-cost ratio (VCR, P < 0.01), benefit-to-cost ratio (BCR, P < 0.01), economic benefit density (EBD, P < 0.01) and irrigation water based on unit benefit (IW_¥_, P < 0.01), and land occupation based on unit benefit (LO_¥_, P < 0.01) among the five rice production modes (**Table 3**). Compared with the CRM mode, the VCR and BCR was only increased in RD and RCF modes, but the EBD was significantly increased and IW_¥ and_ LO_¥_ were significantly decreased in all organic rice production modes indicated that organic rice production increased economic benefits density and improved the economic benefit of crop land and irrigation water use. Compared with the RM mode, the VCR of the RCF, RD and RG modes increased by 84.49%, 74.45% and 18.62%, respectively. The EBD reflects the net profit per unit land area, which was 156.65%, 133.10% and 28.22% higher in the RCF, RD and RG modes, respectively, than that in the RM mode. The EBD of the RCF, RD and RG modes was 2.79, 2.58 and 1.40 times higher, respectively, than that of the RM mode. The IW_¥ of the_ RCF, RD and RG modes was 48.51%, 54.97% and 77.81% of that of the RM mode indicated that the use of irrigation water was reduced by 51.49%, 45.03% and 22.19%, respectively compared with the RM mode based on the unit benefit. There was no significant differences in the LO_¥_ between the RCF and RD mode (P = 0.062), the land occupation was reduced by 57.07% to 60.99% in RCF and RD modes, and that was reduced by 21.88% in the RG mode compared with in the RM mode based on the unit benefit.

### 3.2 Emergy input composition

**Figure 3:**
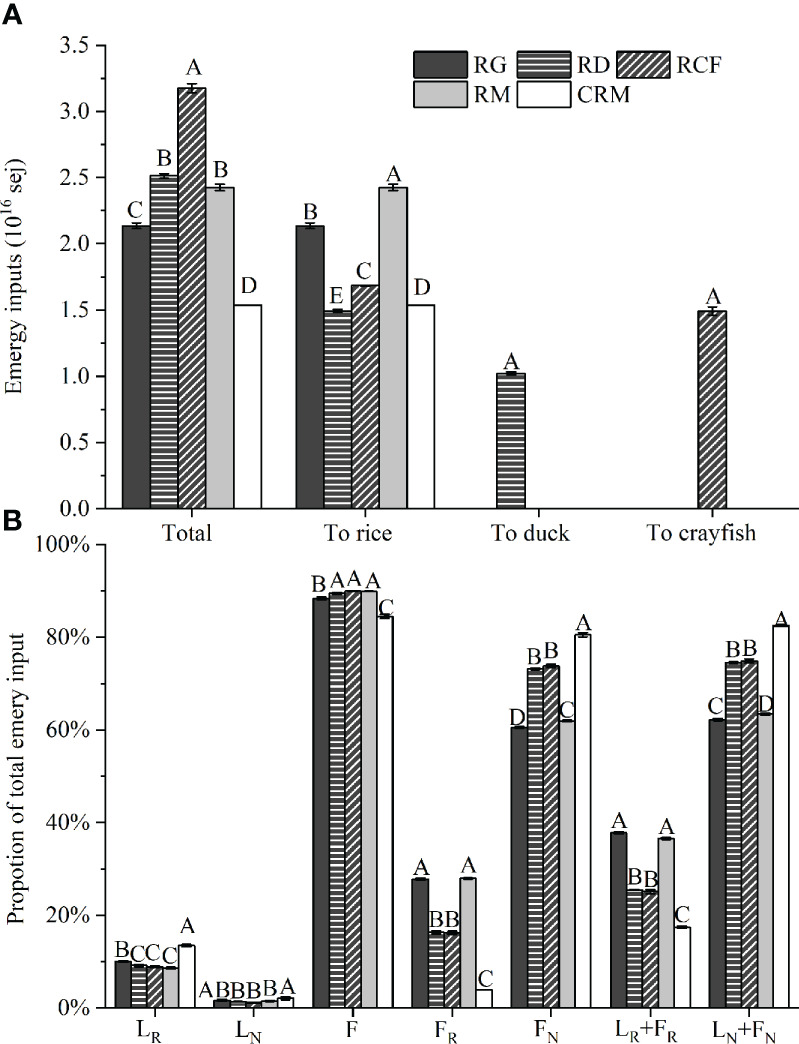
Emergy inputs **(A)** and the composition of total emergy input **(B)** to the five rice production modes. RG, Rice-green manure rotation mode for organic rice production; RD, rice-duck coculture mode for organic rice production; RCF, rice-crayfish coculture mode for organic rice production; RM, rice monoculture mode for organic rice production; CRM, rice monoculture mode for conventional rice production. L_R_, local renewable emergy; L_N_, local nonrenewable resources; F, economic imported resources; F_R_, renewable fraction of purchased inputs; F_N_, nonrenewable fraction of purchased inputs; L_R_ + F_R_, renewable emergy flows; L_N_ + F_N_, nonrenewable emergy flows; In the same item (x-axis label), bars with the same uppercase letters are not significantly different according to the LSD test at P < 0.05.

The emergy inputs and and their components to the five rice production modes are presented in **Figure 3**. There were significant differences in the total emergy input (P < 0.001) among the different five rice production modes. The RCF mode had the highest total emergy input, approximately 3.18E+16 sej/ha/yr, followed by the RD (approximately 2.51E+16 sej/ha/yr), RM (approximately 2.43E+16 sej/ha/yr) and RG (approximately 2.14E+16 sej/ha/yr) modes, and the lowest value was calculated for the CRM mode, at approximately 1.54E+16 sej/ha/yr. Compared the CRM mode, the emergy inputs to rice was only decreased in the RD mode. Since rice was the only product in the RM and RG modes, the emergy inputs to rice in the RM and RG modes were higher than those in the RCF and RD modes. The emergy input to rice in the RCF mode was approximately 1.68E+16 sej/ha/yr, which was higher than that in the RD mode, with a value of 1.49E+16 sej/ha/yr. The proportion of local renewable and nonrenewable resources (L_R_ and L_N_) to the total emergy input were the highest in the CRM mode among all rice production modes due to the lowest total emergy input. Compared with the other organic modes, the proportion of local renewable emergy (L_R_) to the total emergy input was the highest in the RG mode due to the longer cropping cycle and lower total emergy input. The proportion of local nonrenewable resources (L_N_) to the total emergy input was similar among the four organic rice production modes. The economic imported resources (F) accounted for over 80% of the total emergy input in all rice production modes. Although the proportion of F to total emergy input was the lowest in the CRM mode, its proportion of the nonrenewable fraction of purchased inputs (F_N_) and nonrenewable emergy flows (L_N_ + F_N_) to total emergy input were the highest among all rice production modes, which was approximately 79% and 81% respectively. The proportion of the renewable fraction of purchased inputs (F_R_) to the total emergy input was higher in the RG and RM modes than in the RD and RCF modes, which was approximately 28% in the RG and RM modes and 16% in the RD and RCF modes, respectively. Over 70% of the total emergy input to the RD and RCF modes was F_N_, while it accounted for approximately 60% of the total emergy input in the RG and RM modes. The RG mode showed the highest proportion of renewable emergy flows (L_R_ + F_R_) and the lowest L_N_ + F_N_ to total emergy input, at approximately 38% and 62%, respectively. The proportion of L_R_ + F_R_ to total emergy input in the RD mode was equivalent to that in the RCF mode, at approximately 25%, which was the lowest among the organic modes; correspondingly, the proportion of L_N_ + F_N_ to total emergy was highest in the RD and RCF modes, at approximately 75%.

**Figure 4:**
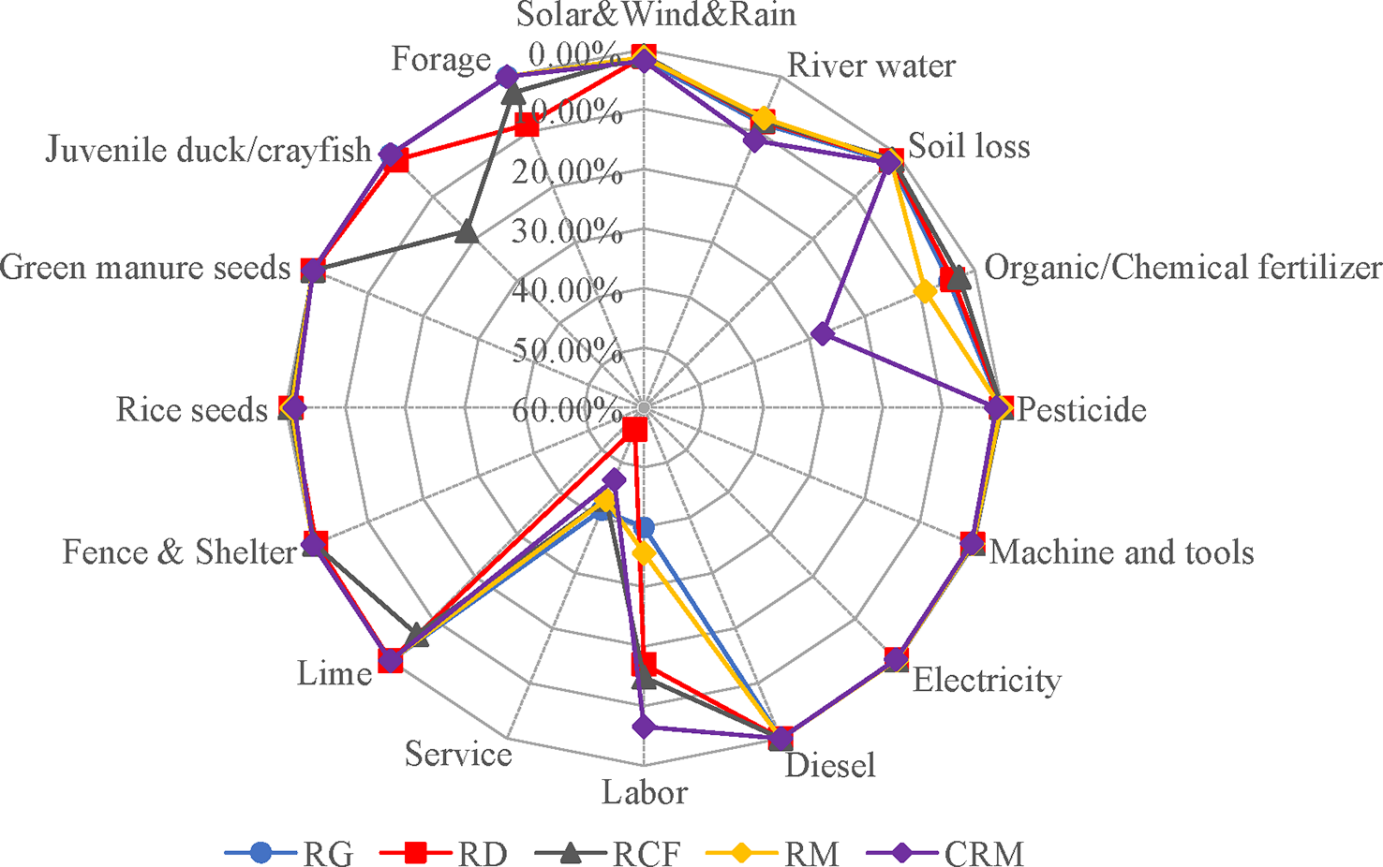
Detailed emergy inputs to the five rice production modes. RD, rice-duck coculture mode for organic rice production; RCF, rice-crayfish coculture mode for organic rice production; RM, rice monoculture mode for organic rice production; CRM, rice monoculture mode for conventional rice production.

Detailed emergy inputs (**Figure 4**) showed that river water (irrigation) was the major free local resource contributing to the organic rice production modes, which accounted for 8.47%, 8.09%, 7.97%, 7.49% and of the total emergy inputs of the RG, RD, RCF, RM and CRM modes, respectively. In the CRM mode, service, chemical fertilizer and labor contributed the major emergy flows from economically imported resources, which accounted for 46.87%, 27.57% and 6.56% of the total emergy input, respectively. In the RM mode, service, labor and organic fertilizer contributed the major emergy flows from economically imported resources, which accounted for 42.49%, 34.91% and 8.84% of the total emergy input, respectively. Compared with the RM mode, growing green manure in the RG mode decreased the amount of organic fertilizer application as well as the total economic imported resource input but did not improve the service and labor inputs, which still accounted for 40.63% and 39.16% of the total emergy input, respectively. In the RD mode, the application of organic fertilizer declined, and the labor input also decreased considerably, but the service input increased in association with the increment in juvenile duck and forage inputs, with a proportion of 55.55% of the total emergy input. In the RCF mode, organic fertilizer and labor inputs also decreased; however, with the large emergy input of juvenile crayfish, lime and forage, the total emery input was significantly increased.

Transformity could be used to measure the efficiency of a product or a production system; the higher the transformity for the same product was, the lower the efficiency of the production system for that product was (Lu et al., 2010). Among the five rice production modes (**Table 4**), RM had the highest transformity of rice followed by RG, RCF, RD and CRM; therefore, the RM mode was the least effective, whereas CRM was the most effective mode for rice production. The transformity of rice with and without labor had an average difference of 39.16% in the RG mode, which was significantly higher than that in the other modes, suggesting that organic rice production in the RG mode depended on labor more than in the other modes. The transformity and specific emergy of rice with and without service had average differences of 49.63%, 45.28%, 46.45%, 42.50% and 40.63% in the RCF, RD, CRM, RM and RG modes, respectively, indicating that organic rice production in all four modes was highly dependent on service and that RCF was the most dependent mode. Between the two rice integrated planting-breeding modes, the transformity of ducks in the RD mode were significantly higher than those of crayfish in the RCF mode, indicating that the production efficiency of ducks was lower than that of crayfish. The differences in the transformitives of ducks and crayfish with and without labor were only approximately 1% for the RD and RCF modes, while those of ducks and crayfish with and without service were 70.57% and 35.63% for the RD and RCF modes, respectively, suggesting that the productions of ducks and crayfish in the RD and RCF modes depended very little on labor and more on service, especially for duck production in the RD mode, which was highly dependent on service.

**Table 4:** Transformatives of the products and their differences between with and without labor and between with and without service from the five rice production modes.

Products	Transformity (10^4^ sej/J)	Difference between with and without labor (%)	Difference between with and without service (%)
Rice in the RG mode	19.07 ± 0.25D	39.93 ± 0.36A	40.63 ± 0.14F
Rice in the RD mode	13.42 ± 0.23F	28.13 ± 0.82C	45.28 ± 0.43D
Rice in the RCF mode	15.49 ± 0.14E	27.32 ± 1.09C	49.63 ± 0.92B
Rice in the RM mode	23.61 ± 0.11C	35.58 ± 0.29B	42.50 ± 0.28E
Rice in the CRM mode	10.45 ± 0.24G	6.65 ± 0.00D	46.45 ± 0.46C
Duck in the RD mode	189.59 ± 4.21A	0.98 ± 0.04E	70.57 ± 0.37A
Crayfish in the RCF mode	169.52 ± 1.09B	1.01 ± 0.06E	35.63 ± 0.10G

RD, rice-duck coculture mode for organic rice production; RCF, rice-crayfish coculture mode for organic rice production; RM, rice monoculture mode for organic rice production; CRM, rice monoculture mode for conventional rice production. The values with different uppercase letters within a column are significantly different according to the LSD test at P < 0.05.

### 3.3 Emergy indices

UEV_E_, UEV_N_ and UEV_¥_ represent the efficiency in converting resources into total available energy content, nutrition density unit and monetary value, respectively. Significant differences in UEV_E_ (P < 0.001), UEV_N_ (P < 0.001) and UEV_¥_ (P < 0.001) were detected among the five rice production modes (**Table 5**). CRM and RCF had the lowest and highest UEV_E_, respectively, thus CRM was the most efficient (and RCF the least efficient) in converting resources to total available energy content. The UEV_E_ was higher in the RD mode than in the RM mode (P < 0.001), suggesting that the efficiency in converting resources to total available energy was higher in the RD mode than in the RM mode. The UEV_N_ did not differ among the RG, RD and RCF modes (P = 0.216, P = 0.593 and P = 0.094) but was significantly higher (P < 0.001) than that in the CRM mode and lower (P < 0.001) than that in the RM mode, indicating that the efficiency in converting resources to nutrition density units was the highest in the CRM mode and the lowest in the RM mode. The RM and CRM modes showed the highest and lowest UEV_¥,_ respectively, indicating the lowest and highest efficiency in terms of the monetary value was generated in the RM and CRM modes, respectively, among the five modes. The RG and RCF modes did not differ (P = 0.072) in the value of UEV_¥_, suggesting that efficiency in converting resources to monetary value in the RG mode was equivalent to that in the RCF mode.

**Table 5:** Comparison of emergy indices for the five rice production modes.

Emergy indices	RG	RD	RCF	RM	CRM
UEV_E_ (10^4^ sej/J) ^a^	19.07 ± 0.25D	21.68 ± 0.32C	27.18 ± 0.33A	23.61 ± 0.11B	10.45 ± 0.14E
UEV_N_ (10^11^ sej/NDU) ^b^	11.78 ± 0.16B	12.23 ± 0.16B	11.79 ± 0.07B	14.59 ± 0.07A	6.46 ± 0.09C
UEV_¥_ (10^10^ sej/¥) ^c^	35.11 ± 0.46B	28.89 ± 0.36C	34.14 ± 0.20B	41.47 ± 0.73A	25.80 ± 0.40D
%R (%)	38.54 ± 0.23A	25.70 ± 0.14C	25.29 ± 0.42C	37.27 ± 0.22B	17.44 ± 0.21D
EYR	1.13 ± 0.00B	1.12 ± 0.00C	1.11 ± 0.00C	1.12 ± 0.00C	1.18 ± 0.01A
ELR	1.60 ± 0.02C	2.89 ± 0.02B	2.96 ± 0.07B	1.68 ± 0.02C	4.74 ± 0.07A
ESI	0.71 ± 0.01A	0.39 ± 0.00C	0.38 ± 0.01C	0.66 ± 0.01B	0.25 ± 0.00D

RD, rice-duck coculture mode for organic rice production; RCF, rice-crayfish coculture mode for organic rice production; RM, rice monoculture mode for organic rice production; CRM, rice monoculture mode for conventional rice production.^a^UEV_E_ = total emergy input/total available energy content.^b^UEV_N =_ total emergy input/total nutrition density unit. The calculation of nutrition density unit (NDU) was based on Xu et al. (2022). The amount of essential fatty acids, protein, and fiber in 100 g of rice, duck and crayfish come from Yang (2018).^c^UEV_¥_ = total emergy input/total monetary value. The total monetary value refers to the money received when these products are sold from by the farm to the retailer. The values with different uppercase letters within a row are significantly different according to the LSD test at P < 0.05.

The renewable fraction (%R) represents the proportion of renewable resources to total emergy inputs in a production system, and a low %R indicates the low sustainability of a production system. As shown in **Table 5**, there were significant differences in the %R values among organic production modes (P < 0.001). The %R of the RG mode was the highest followed by those of the RD and RCF and that of the CRM was the lowest. The higher %R of the organic rice production modes was mainly due to the placement of a large number of nonrenewable resources, such as fertilizers and pesticides application in the CRM mode. Compared with the RM mode, the replacement of a large amount of organic fertilizer in the RM mode also led to the increase of R% in the RG mode. However, although the inputs of organic fertilizer, labor and pesticides in the RD and RCF modes were largely decreased compared with those in the RM mode, the increased service input for the purchase of juvenile duck and crayfish and forage significantly increased the proportion of nonrenewable resources.A high emergy yield ratio (EYR) indicates a high ability of the system to exploit free natural resources (Odum, 1996; Zhang et al., 2011). As presented in **Table 5**, the ELR differed significantly with the five rice production modes (P < 0.001). There was no significant difference in the EYR among the RD, RCF and RM modes (P = 0.195), while those of the RG and CRM modes were higher, suggesting the higher ability to exploit local free resources in the RG and CRM modes.The environmental loading ratio (ELR) reflects the input level of nonrenewable resources. Based on the classification by Brown and Ulgiati, 2004, an ELR higher than 10 indicates a much higher environmental impact, an ELR lower than 2 indicates a low environmental impact, and an ELR between 2 and 10 indicates a moderate environmental impact. The ELR significantly differed with rice production modes (P < 0.001, **Table 5**). The ELR of the CRM was the highest among the five rice production modes that indicated the CRM mode could cause a greater environmental pressure than other organic rice production modes. There was no significant difference in the ELR between the RD and RCF modes (P = 0.330), with a value of approximately 2.90, which was higher than the ELR value of the RG and RM modes, indicating a moderate environmental impact. These findings could be due to the higher service input in the RD and RCF modes, causing a higher environmental pressure. An emergy sustainability index (ESI) below 1 means that the system achieves its production by generating a higher environmental pressure, while a living and more sustainable system corresponding to an ESI between 1 and 10 (Hou et al., 2021). All five rice production modes were highly dependent on purchased emergy inputs, and the ESI of all five modes was lower than 1 (**Table 5**). Significant differences in the ESI were detected among the five modes (P < 0.001). The ESI of the CRM, with a value of approximately 0.25, was the lowest among the five rice production modes, indicating the highest environmental pressure and the worst sustainability. Compared with the RM mode, the ESI increased in the RG mode but decreased in the RD and RCF modes, indicating that the rice-green manure rotation improved sustainability, while the association of rice and duck or crayfish production increased the environmental pressure on organic rice culture compared with the rice monoculture.

### 3.4 Scenario simulation and optimization

Although the RD and RCF modes showed higher efficiency in converting resources to total nutrition density units and monetary value and greater economic viability, they caused a higher environmental pressure with lower emergy utilization efficiency and ESI due to the higher service input in these two modes. Therefore, scenario simulation was conducted to determine whether the emergy utilization efficiency and ESI could be improved. According to the characteristics of the emergy input structure, starting with the key points affecting the ecological performance of the system, the performances of the key indicators of the RD and RCF modes of the system under the situation of different technology improvements were simulated (**Table 6**). The cropping cycle of rice-duck coculture did not conflict with green manure growing; therefore, green manure growing could combine with rice-duck coculture to replace the organic fertilizer input in the RD mode. Compared with the RD mode, if rice-duck coculture is combined with green manure growing, it would significantly decrease (P = 0.002) the UEV_E_ but not significantly affect the ESI (P = 0.718), indicating that the emergy utilization efficiency would increase but the sustainability would not be improved. In the RD mode, forage accounted for 8.63% of the total emergy input and was the major fraction (23.41%) of service input. Rice barns are a kind of nutritious by-products in rice processing that could be used to replace the purchased forage in the RD mode, as reported by Pirdashti et al. (2015). If the forage is replaced by rice barns in the RD mode, the UEV_E_ and ESI would reach 1.71E+05 sej/J and 0.50, which indicates that the emergy utilization efficiency and ESI would be increased by 21.62% and 24.00% compared to the current level, respectively. Juvenile crayfish accounted for 17.56% and 17.07% of the total emergy and service input, respectively. According to Wang et al. (2020), the crayfish yield could reach 1035 to 1455 kg/ha when 750 kg/ha adult crayfish was input under different feeding methods in paddy fields. Therefore, reserving 20% of the crayfish yield (271 kg/ha) could meet the demand of crayfish input (450 kg/ha) in the next season in the RCF mode. After reserving 20% of the crayfish yield to replace the crayfish input in the next season, the emergy utilization efficiency and ESI in the RCF mode would be 27.37% and 24.49% higher than the current level, respectively.

**Table 6:** Scenarios description and simulation results.

Scenarios	Description	Original		Optimized	
		UEV_E_ (10^4^ sej/J)	ESI	UEV_E_ (10^4^ sej/J)	ESI
1	Rice-duck coculture combined with green manure growing	21.68 ± 0.32	0.39 ± 0.00	19.78 ± 0.33*	0.39 ± 0.01
2	Replacement of forage with rice barn in rice-duck coculture	21.68 ± 0.32	0.39 ± 0.00	17.07 ± 0.21*	0.51 ± 0.00*
3	Reserve 20% crayfish yield to replace the crayfish input in next season	27.18 ± 0.33	0.38 ± 0.01	19.68 ± 0.14*	0.50 ± 0.01*

The values with * indicate there are significant differences between the original and optimized values according to the LSD test at P < 0.05.

## 4 Discussion

### 4.1 Driving force analysis of the formation of organic rice production modes

According to the Sustainable Development Goals (SDG), by 2030, the global agricultural system is supposed to make sustainable food production accessible to all people (Arunrat et al., 2021). Organic rice cultivation is encouraged owing to it is expected to lower risks from eliminating chemical inputs and reduce environmental impacts, including ecosystem degradation and global warming (Mungkung et al., 2020). It has been reported that the demand for organic rice in the international market is increasing and supposed to continue growing (Declaro-Ruedas, 2019). Optimal pest control and sufficient fertilization are fundamental for rice productivity. As the most basic organic rice production mode, the RM mode requires large investments in organic fertilizer and labor to ensure the rice yield due to difficulties in plant nutrient management and a lack of effective pest management options (especially for weed management). In this study, labor and organic fertilizer were the major costs in the RM mode, accounting for 52.81% and 23.27% of the total cost, respectively. Compared with the RM mode, the RG mode decreased organic fertilizer by 52.81% and increased rice yield by 8.99%. This result was consistent with the conclusion proposed by Hazra et al. (2018), who reported that the integrated application of different sources of organic inputs could increase the rice yield compared to the use of only one organic source at the similarly recommended fertilizer rate. The driving force for the organic rice production shift from the RM mode to the RG mode could be explained by lower input and higher profit. However, the RG mode did not improve the labor input, which accounted for 52.81% of the total cost. Over the past decade in China, as an important factor of agricultural production, the rural labor force has gradually changed from having a surplus to a scarcity with the transformation of the population structure and the in-depth promotion of industrialization and urbanization, and its structure is facing great challenges such as aging, feminization and part-time employment (Liu and Zhou, 2022). Therefore, the demand for the labor force in agricultural production is tight, the cost has increased, and the production mode of organic rice, which requires a large amount of labor input, also faces great challenges.

Rice integrated planting-breeding systems create a yield-increasing model of the integrated utilization of paddy resources for rice production and waterfowl or aquatic animal breeding, and a mutually beneficial situation is established for both rice growing and waterfowl or aquatic animal breeding in this system (Pirdashti et al., 2015; Xu et al., 2019; Hou et al., 2021). In the RD and RCF modes, ducks and crayfish could prey on weed seedlings and pests (Pirdashti et al., 2015; Zhou et al., 2022), which would reduce the inputs needed for weed and pest control, and the wastes of duck and crayfish and forage over application could increase the nutrient supply for rice and reduce its dependence on fertilizers (Clavero et al., 2015; Xi and Qin, 2020). Hou et al. (2021) found that rice–crayfish had approximately 31% of IW_¥_ and 14–18% of LO_¥_ compared with rice monoculture, indicating that rice–crayfish systems improved the economic benefit of crop land and irrigation water use in conventional rice production system. Similarly, our study indicated that the use of irrigation water and crop land in the RCF mode was reduced by 51.49% and 60.99%, respectively, and those was reduced by 45.03% and 57.07%, respectively in the RD mode compared with in the RM mode based on the unit benefit in organic production mode. In this study, 53.55% and 58.33% of organic fertilizer and 50.03% and 44.58% of labor were decreased in the RD and RCF modes, respectively, compared with the RM mode. The RD and RCF modes not only did not reduce the rice yield but also increased the production of duck and crayfish. Therefore, the profits of the RD and RCF modes were much higher than that of the RM mode. The EBD of the RCF and RD modes was 2.79 and 2.58 times higher, respectively, than that of the RM mode. The lower labor input and higher profit could be the reason that the RD and RCF modes were more favored by farmers than the RM mode in organic rice production. Although the annual average costs of the RG, RD and RCF mode were similar, there was a one-time heavy upfront capital investment in the first year in the RD and RCF modes due the costs of shelter and fencing in the RD mode and the costs of fencing and breeding ditch construction in the RCF mode. Furthermore, the RD or RCF mode requires not only rice planting technology but also breeding technology for ducks or crayfish. These reasons could explain why the planting area of the RD and RCF modes was lower than that of the RG mode.

### 4.2 Sustainability and future development strategy

Sustainability comparisons between organic rice and conventional (based on chemical fertilizer and pesticide inputs) rice production or integrated planting-breeding modes and conventional rice production have been widely reported, but sustainability comparisons among different organic rice production modes are still rare. As described by Xu et al. (2019), the total emergy input of the rice-crab coculture system was higher than that of the conventional rice monoculture in the Liaohe River basin, China. Hou et al. (2021) also obtained similar conclusions in studies of rice-crayfish coculture systems compared with conventional rice monoculture and double rice cropping systems. However, Xi and Qin (2009) reported that the total emergy input of organic rice-duck mutualism systems was lower than that of conventional rice-wheat rotation systems. Our results showed that the conventional rice monoculture mode had the lowest total emergy input, and among the four organic rice production modes, the total emergy inputs of rice integrated planting-breeding modes (i.e., the RCF and RD modes) were higher than those of the RM mode, while that of the RG mode was the lowest. Since rice was the only product in the RM and RG modes, the emergy inputs to rice in the RM and RG modes were higher than those in the RCF and RD modes. A lower UEV can be regarded as an indicator of the higher efficiency of a system when comparing systems with the same product (Zhang et al., 2011). In our study, with the lowest total emergy input and the highest rice yield, the CRM mode showed the highest ecological efficiency in converting resources to total available energy content, nutrition density unit and monetary value among the five rice production modes. The two rice-integrated planting-breeding modes (RCF and RD) not only had a rice yield that was not significantly less than that of the RG mode but also had the additional output of ducks and crayfish. However, due to the higher total emery inputs in the RCF and RD modes, the efficiency in converting resources to the total available energy content of the RCF and RD modes was lower than that of the RG mode, and that of the RCF mode was even lower than that of the RM mode, based on the UEV_E_ performance among the four organic rice production modes. As far as we know, only two articles based on EMA have compared the UEVs among rice production modes. Xu et al. (2019) and Hou et al. (2021) suggested that rice-crab coculture and rice-crayfish coculture demonstrated a lower efficiency in UEV_E_ but a higher efficiency in UEV_¥_ compared with conventional rice monoculture. In addition to the additional output of crab and crayfish, the higher efficiency in UEV_¥_ in rice-crab and rice-crayfish cocultures was attributed to the higher rice yield and price than that of the conventional rice monoculture in their studies. The lower pesticide and chemical fertilizer input in rice-crab and rice-crayfish cocultures explained the high price of rice sold to customers (Xu et al., 2019; Hou et al., 2021). In this case, there was no significant difference in the rice yield among the RG, RD and RCF modes, and the rice price was the same. Although there was additional outputs of duck and crayfish in the RD and RCF modes, the efficiency in terms of monetary value generated by the RG mode was only lower than that of the RD mode, and the efficiency in converting resources to nutrition density units did not differ among the RG, RD and RCF modes.

A sustainable production system should consume less renewable and nonrenewable resources at the same time to produce a given quantity and of quality products (Xu et al., 2019). The previous studies reported that the proportion of renewable resources to total emergy inputs (%R) in the conventional rice monoculture was between 26% and 29% (Xi and Qin, 2009; Li et al., 2018; Hou et al., 2021). Our results demonstrated that the %R in the CRM was only 17% which was lower than those of in the other organic rice production modes due to the large input of non-renewable chemical fertilizers and pesticides. The %R of the RG mode was the highest among the four organic rice production modes due to the replacement of a large amount of organic fertilizer in the RM mode. However, although the inputs of organic fertilizer, labor and pesticides in the RD and RCF modes were largely decreased compared with those in the RM mode, the increased service input for the purchase of juvenile duck and crayfish and forage significantly increased the proportion of nonrenewable resources. Among the four organic rice production modes, the RG mode had the lowest ELR, and the highest ESI indicated that the RG mode was the most sustainable organic production mode. Xi and Qin (2009) demonstrated that the ESI of a rice-duck farming system was 8.7 times greater than that of a conventional rice-wheat rotation system in Shanghai; however, the feedback yield energy reused in the system was also included in the emergy index calculation process, which might lead to the overestimation of the ESI of the rice-duck mutualism system. Li et al. (2018) conducted emergy evaluations of rice-duck farming and conventional rice monocropping systems in the Taihu Lake catchment and indicated that the ESI of rice-duck farming was 2.21 and that of rice monocropping was 1.92. However, the ESI of conventional rice monoculture, which was highly dependent on purchased emergy inputs, should not exceed 1 according to the studies of Xu et al. (2019) and Hou et al. (2021). In the present study, the ESI was only 0.25 in the CRM mode, which was lower than those of in the four organic rice production modes indicated that the conventional mode has higher environmental pressure and lower sustainability than the organic modes. Hou et al. (2021) assessed the environmental pressure of rice-crayfish coculture and conventional rice monoculture in Jianghan Plain and demonstrated that the ESI of rice-crayfish coculture was lower than that of rice monoculture. A similar conclusion was obtained in this study that the ESI of the RCF mode was lower than that of the RM mode under organic rice production. It is believed that plant-diversified farming practices can contribute to ecological intensification of agriculture (Gurr et al., 2016; Zhao et al., 2016; Redlich et al., 2018). A study by Wan et al. (2019) demonstrated that compared to the mono-rice farming for conventional rice production, rice-fish co-culture decreased insect and weed abundance, increased invertebrate predator abundance and reduced the need for pesticide and produced an higher average economic values, and confirmed that rice-fish co-culture can be an effective form of ecological intensification, incorporating and contributing ecosystem services in agricultural production and increasing sustainability accordingly. Similarly, our results indicated that compared to the RM mode, the RG, RD and RCF modes promoted ecological intensification of organic rice production by reducing organic fertilizer input and increasing rice yield and economic benefits. Meanwhile, the RG mode had higher emergy utilization efficiency and the higher renewable fraction and improved sustainability of organic rice production than those of RM mode. However, the RD and RCF modes imposed higher environmental pressure with a lower renewable fraction and emergy sustainability index than those in the RM mode.

Labor and services are important input flows to a process because they represent the total emergy that supports workers’ life, transportation and education throughout the supply chain process, as well as the social infrastructure that supports the process itself (Ulgiati and Brown, 2014). In this study, labor and service were the major emergy inputs in the four organic rice production modes, accounting for 79.79%, 72.39%, 59.74%, and 77.40% of the total emergy inputs in the RG, RD, RCF and RM modes, respectively, which were higher than the related conventional rice integrated planting-breeding and rice monoculture modes (Xi and Qin, 2009; Li et al., 2018; Hou et al., 2021). Our field investigation showed that to ensure the yield of rice, the input amount and cost of organic fertilizer in organic rice production were much higher than those of chemical fertilizer in conventional rice production, and due to a lack of effective weed management options, the weed control of organic rice mainly depended on hand weeding, which led to an increase in the inputs of service and labor in organic rice production. If a system becomes self-sufficient through internal recycling, then the amount of imported nonrenewable parts can be reduced, and the total services can be subsequently decreased (Xu et al., 2019). The results of scenario simulation and optimization showed that the rice-duck coculture combined with green manure growing would significantly increase the emergy utilization efficiency of the RD mode but would not improve its sustainability. If forage is replaced by rice barns in the RD mode, the emergy utilization efficiency will improve which would be even higher than that in the RG mode, and ESI will increase by 24.00% compared to the current level, respectively. Similarly, after reserving 20% crayfish of the yield to replace the crayfish input in the next season, the emergy utilization efficiency and ESI in the RCF mode will be 27.37% and 24.49% higher than the current level, respectively.

## 5 Conclusion

This study evaluated the economic viability and environmental and sustainability performances of the four dominant organic (RG, RD, RCF and RM) and one conventional (CRM) rice production modes in Jiangsu Province. Compared with the CRM mode, organic rice production increased economic benefits density and improved the economic benefit of crop land and irrigation water use. With the lowest total emergy input and the highest rice yield, the CRM mode showed the highest ecological efficiency in converting resources to total available energy content, nutrition density unit and monetary value among the five rice production modes. However, the RCM mode showed higher environmental pressure and lower sustainability than the four organic modes due to the larger proportion of nonrenewable emergy input. The RM mode was the most uneconomic organic rice production mode with the highest cost input and the lowest product output but showed a relatively higher sustainability due to the higher proportion of renewable resources to total emergy inputs. The RD and RCF modes had better economic benefits and higher efficiency in converting resources to nutrition density units than did the RM mode; however, they showed higher environmental pressure and lower sustainability due to the higher service inputs. Considering the economic and environmental impacts, RG model had high economic feasibility and the best agricultural production sustainability, which is possible to considerably improve the economic welfare of local farmers without increasing the environmental burden. The large labor input is the primary factor limiting the development of rice-green manure rotation; therefore, efficient weed management measures (such as bioherbicides, integrated weed management based on cultivation practices including film mulching, tillage, irrigation, ect.) that cast off or greatly reduce labor costs should be developed to further promote rice-green manure rotation for organic rice production.

## Data availability statement

The original contributions presented in the study are included in the article/**Supplementary Material**. Further inquiries can be directed to the corresponding author.

## Author contributions

QD contributed to the conception of the study. PG, HW, and ED performed the field survey. GS, QX, ZD, and WW contributed significantly to analysis and manuscript preparation. PG performed the data analyses and wrote the manuscript. GS, QX, ZD, and WW helped perform the analysis with constructive discussions. All authors contributed to the article and approved the submitted version.
