# Supplementary material for: Integrated emergy and economic evaluation of the dominant organic rice production systems in Jiangsu province, China
Source: Front Plant Sci. 2023 Mar 24;14:1107880. doi: 10.3389/fpls.2023.1107880 (PMC10081491; doi:10.3389/fpls.2023.1107880)
Supplement: Supplementary file 1 [file Table_1.docx]

Table A

Emergy analysis of rice-green manure rotation mode for organic rice production (/ha/yr).

| **NO.** | **Item** | **Raw data** | **RNF^a^** | **UEV^a^ (sej/ unit)** | **Emergy (sej)** |
| --- | --- | --- | --- | --- | --- |
|  | Free local renewable resources (L_R_) |  |  |  |  |
| **1** | Solar energy (J/ha) | 3.64E+13 | 1.00 | 1.00 | 3.64E+13 |
| **2** | Wind energy (J/ha) | 7.39E+09 | 1.00 | 8.00E+02 | 5.91E+12 |
| **3** | Rain chemical energy (J/ha) | 4.60E+10 | 1.00 | 7.00E+03 | 3.22E+14 |
| **4** | River water (Chemical, J/ha) | 2.73E+10 | 1.00 | 6.50E+04 | 1.77E+15 |
|  | Free local nonrenewable resources (L_N_) |  |  |  |  |
| **5** | Energy of net soil loss (J/ha) | 3.74E+09 | 0.00 | 9.40E+04 | 3.51E+14 |
|  | Economic imported resources (F) |  |  |  |  |
| **6** | Organic fertilizer^b^ (J/ha) | 3.68E+10 | 0.68 | 2.70E+04 | 9.95E+14 |
| **7** | Pesticide (g/ha) | 2.42E+03 | 0.00 | 1.89E+10 | 4.57E+13 |
| **8** | Machine and tools (g/ha) | 1.09E+04 | 0.00 | 8.57E+09 | 9.30E+13 |
| **9** | Electricity (J/ha) | 2.25E+08 | 0.09 | 2.04E+05 | 4.60E+13 |
| **10** | Diesel (g/ha) | 6.28E+04 | 0.00 | 4.09E+04 | 2.57E+09 |
| **11** | Labor^c^ (yr/ha) | 1.68E-01 | 0.60 | 4.98E+16 | 8.36E+15 |
| **12** | Services (¥/ha) | 1.15E+04 | 0.00 | 7.54E+11 | 8.67E+15 |
| **13** | Rice seeds (J/ha) | 9.12E+08 | 1.00 | 2.55E+05 | 2.33E+14 |
| **14** | Green manure seed (g/ha) | 9.57E+07 | 1.00 | 2.70E+04 | 2.58E+12 |
|  | Feedback yield energy in the system^d^ |  |  |  |  |
| **15** | green manure (J/ha) | 4.71E+10 | *0.48^e^* | 4.70E+03 | 0.00 |
| **16** | Nitrogen fixation (g/ha) | 6.31E+04 | *0.48^e^* | 3.80E+09 | 0.00 |
| **17** | Straw (J/ha) | 5.05E+10 | 0.37 | 5.99E+03 | 0.00 |
| **18** | Yield of rice (J/ha) | 1.10E+11 |  | 1*.91E+05^e^* |  |

^a^ RNF and UEV reference for respective row numbers (converted to 12.0E+24 sej/year baseline): 1, 2, 3, Brown and Ulgiati (2016); 4, 5 Odum (1996); 6 Yang and Chen (2014); 7, 8, 9, 10, 13 Lan et al. (2002); 11, 12 Lou et al. (2015); 14 Yang et al. (2017); 15, 16 Xi and Qin (2020); 17, Wang et al. (2017).

^b^ The organic fertilizer contained 45.4% organic matter, 2.0% N, 2.9% P_2_O_5_, 1.2% K_2_O, and 29.1% water..

^c^ The raw data of 1.68E-01 year/ha corresponds to 15112 ¥/ha.

^d^ According to emergy theory by Brown and Ulgiati (1997), feedback yield energy are reused in the system, therefore, the emergy values are zero.

^e^ Italic: Calculated from this study.

Table B

Emergy analysis of rice-duck coculture mode for organic rice production (/ha/yr).

| **NO.** | **Item** | **Raw data** | **RNF^a^** | **UEV^a^ (sej/ unit)** | **Emergy (sej)** |
| --- | --- | --- | --- | --- | --- |
|  | Free local renewable resources (L_R_) |  |  |  |  |
| **1** | Solar energy (J/ha) | 1.63E+13 | 1.00 | 1 | 1.63E+13 |
| **2** | Wind energy (J/ha) | 2.70E+09 | 1.00 | 8.00E+02 | 2.16E+12 |
| **3** | Rain chemical energy (J/ha) | 3.83E+10 | 1.00 | 7.00E+03 | 2.68E+14 |
| **4** | River water (Chemical, J/ha) | 3.10E+10 | 1.00 | 6.50E+04 | 2.01E+15 |
|  | Free local nonrenewable resources (L_N_) |  |  |  |  |
| **5** | Energy of net soil loss (J/ha) | 3.72E+09 | 0.00 | 9.40E+04 | 3.50E+14 |
|  | Economic imported resources (F) |  |  |  |  |
| **6** | Organic fertilizer^b^ (J/ha) | 3.69E+10 | 0.68 | 2.70E+04 | 9.96E+14 |
| **7** | Pesticide (g/ha) | 1.35E+03 | 0.00 | 1.89E+10 | 2.54E+13 |
| **8** | Machine and tools (g/ha) | 1.15E+04 | 0.00 | 8.57E+09 | 9.86E+13 |
| **9** | Electricity (J/ha) | 2.56E+08 | 0.09 | 2.04E+05 | 5.22E+13 |
| **10** | Diesel (g/ha) | 6.29E+04 | 0.00 | 4.09E+04 | 2.57E+09 |
| **11** | Labor^c^ (yr/ha) | 8.50E-02 | 0.60 | 4.98E+16 | 4.23E+15 |
| **12** | Services (¥/ha) | 1.85E+04 | 0.00 | 7.54E+11 | 1.40E+16 |
| **13** | Shelter and fence |  |  |  |  |
| **a** | Wood (g/ha) | 2.81E+05 | 1.00 | 5.14E+08 | 1.44E+14 |
| **b** | Plastic film (g/ha) | 2.37E+04 | 0.00 | 2.88E+08 | 6.82E+12 |
| **14** | Rice seeds J/ha | 9.12E+08 | 1.00 | 2.55E+05 | 2.33E+14 |
| **15** | Juvenile duck J/ha | 1.97E+08 | 0.20 | 1.70E+06 | 3.35E+14 |
| **16** | Forage (g/ha) | 1.28E+06 | 0.20 | 1.70E+09 | 2.17E+15 |
|  | Feedback yield energy in the system^d^ |  |  |  |  |
| **17** | Straw (J/ha) | 4.45E+10 | 0.37 | 5.99E+03 | 0.00 |
| **18** | Duck feces (g/ha) | 1.19E+05 | 0.20 | - | 0.00 |
|  | Yield of rice (J/ha) | 1.09E+11 |  | *1.34E+05^e^* |  |
|  | Yield of duck (J/ha) | 5.39E+09 |  | *1.90E+06^e^* |  |

^a^ RNF and UEV reference for respective row numbers (converted to 12.0E+24 sej/year baseline): 1, 2, 3, Brown and Ulgiati (2016); 4, 5, 13b Odum (1996); 6 Yang and Chen (2014); 7, 8, 9, 10, 13a, 14 Lan et al. (2002); 11, 12 Lou et al. (2015); 14 Yang et al. (2017); 15, 16 Xi and Qin (2020); 17, Wang et al. (2017).

^b^ The organic fertilizer contained 45.4% organic matter, 2.0% N, 2.9% P_2_O_5_, 1.2% K_2_O, and 29.1% water.

^c^ The raw data of 8.50E-02 year/ha corresponds to 7650 ¥/ha.

^d^ According to emergy theory by Brown and Ulgiati (1997), feedback yield energy are reused in the system, therefore, the emergy values are zero.

^e^ Italic: Calculated from this study.

Table C

Emergy analysis of rice-crayfish coculture mode for organic rice production (/ha/yr).

| **NO.** | **Item** | **Raw data** | **RNF^a^** | **UEV^a^ (sej/ unit)** | **Emergy (sej)** |
| --- | --- | --- | --- | --- | --- |
|  | Free local renewable resources (L_R_) |  |  |  |  |
| **1** | Solar energy (J/ha) | 2.08E+13 | 1.00 | 1 | 2.08E+13 |
| **2** | Wind energy (J/ha) | 4.89E+09 | 1.00 | 8.00E+02 | 3.91E+12 |
| **3** | Rain chemical energy (J/ha) | 4.17E+10 | 1.00 | 7.00E+03 | 2.92E+14 |
| **4** | River water (Chemical, J/ha) | 3.87E+10 | 1.00 | 6.50E+04 | 2.51E+15 |
|  | Free local nonrenewable resources (L_N_) |  |  |  |  |
| **5** | Energy of net soil loss (J/ha) | 3.84E+09 | 0.00 | 9.40E+04 | 3.61E+14 |
|  | Economic imported resources (F) |  |  |  |  |
| **6** | Organic fertilizer^b^ (J/ha) | 3.31E+10 | 0.68 | 2.70E+04 | 8.94E+14 |
| **7** | Pesticide (g/ha) | 1.35E+03 | 0.00 | 1.89E+10 | 2.54E+13 |
| **8** | Machine and tools (g/ha) | 1.36E+04 | 0.00 | 8.57E+09 | 1.17E+14 |
| **9** | Electricity (J/ha) | 3.19E+08 | 0.09 | 2.04E+05 | 6.51E+13 |
| **10** | Diesel (g/ha) | 8.11E+04 | 0.00 | 4.09E+04 | 3.32E+09 |
| **11** | Labor^c^ (yr/ha) | 9.41E-02 | 0.60 | 4.98E+16 | 4.69E+15 |
| **12** | Services (¥/ha) | 1.81E+04 | 0.00 | 7.54E+11 | 1.37E+16 |
| **13** | Lime (g/ha) | 1.55E+06 | 0.05 | 1.27E+09 | 1.97E+15 |
| **14** | Fence |  |  |  |  |
| **a** | Wood (g/ha) | 1.69E+05 | 1.00 | 5.14E+08 | 8.70E+13 |
| **b** | Plastic film (g/ha) | 1.48E+04 | 0.00 | 2.88E+08 | 4.26E+12 |
| **15** | Rice seeds (J/ha) | 8.21E+08 | 1.00 | 2.55E+05 | 2.09E+14 |
| **16** | Juvenile crayfish (J/ha) | 2.90E+09 | 0.20 | 1.96E+06 | 5.69E+15 |
| **17** | Forage (g/ha) | 5.40E+05 | 0.20 | 1.70E+09 | 9.18E+14 |
|  | Feedback yield energy in the system^c^ |  |  |  |  |
| **18** | Straw (J/ha) | 4.15E+10 | 0.37 | 5.99E+03 | 0 |
| **19** | Crayfish feces (g/ha) | 9.56E+04 | 0.20 | - | 0 |
|  | Yield of rice (J/ha) | 1.07E+11 |  | *1.55E+05^e^* |  |
|  | Yield of crayfish (J/ha) | 8.80E+09 |  | *1.70E+06^e^* |  |

^a^ RNF and UEV reference for respective row numbers (converted to 12.0E+24 sej/year baseline): 1, 2, 3, Brown and Ulgiati (2016); 4, 5, 14b Odum (1996); 6 Yang and Chen (2014); 7, 8, 9, 10, 14a, 15 Lan et al. (2002); 11, 12 Lou et al. (2015); 13 Cavalett et al. (2006); 16, 17 Li et al. (2011); 18, Wang et al. (2017).

^b^ The organic fertilizer contained 45.4% organic matter, 2.0% N, 2.9% P_2_O_5_, 1.2% K_2_O, and 29.1% water.

^c^ The raw data of 9.41E-02 year/ha corresponds to 8470 ¥/ha.

^d^ According to emergy theory by Brown and Ulgiati (1997), feedback yield energy are reused in the system, therefore, the emergy values are zero.

^e^ Italic: Calculated from this study.

Table D

Emergy analysis of rice monoculture mode for organic rice production (/ha/yr).

| **NO.** | **Item** | **Raw data** | **RNF^a^** | **UEV^a^ (sej/ unit)** | **Emergy (sej)** |
| --- | --- | --- | --- | --- | --- |
|  | Free local renewable resources (L_R_) |  |  |  |  |
| **1** | Solar energy (J/ha) | 1.61E+13 | 1.00 | 1 | 1.61E+13 |
| **2** | Wind energy (J/ha) | 4.93E+09 | 1.00 | 8.00E+02 | 3.94E+12 |
| **3** | Rain chemical energy (J/ha) | 4.16E+10 | 1.00 | 7.00E+03 | 2.92E+14 |
| **4** | River water (Chemical) (J/ha) | 2.74E+10 | 1.00 | 6.50E+04 | 1.78E+15 |
|  | Free local nonrenewable resources (L_N_) |  |  |  |  |
| **5** | Energy of net soil loss (J/ha) | 3.86E+09 | 0.00 | 9.40E+04 | 3.62E+14 |
|  | Economic imported resources (F) |  |  |  |  |
| **6** | Organic fertilizer^b^ (J/ha) | 7.94E+10 | 0.68 | 2.70E+04 | 2.14E+15 |
| **7** | Pesticide (g/ha) | 2.69E+03 | 0.00 | 1.89E+10 | 5.08E+13 |
| **8** | Machine and tools g | 1.09E+04 | 0.00 | 8.57E+09 | 9.32E+13 |
| **9** | Electricity (J/ha) | 2.26E+08 | 0.09 | 2.04E+05 | 4.62E+13 |
| **10** | Diesel (g/ha) | 6.31E+04 | 0.00 | 4.09E+04 | 2.58E+09 |
| **11** | Labor^c^ (yr/ha) | 1.70E-01 | 0.60 | 4.98E+16 | 8.47E+15 |
| **12** | Services (¥/ha) | 1.37E+04 | 0.00 | 7.54E+11 | 1.03E+16 |
| **13** | Rice seeds (J/ha) | 9.12E+08 | 1.00 | 2.55E+05 | 2.33E+14 |
|  | Feedback yield energy in the system^d^ |  |  |  |  |
| **14** | Straw (J/ha) | 6.25E+10 | 0.37 | 5.99E+03 | 0.00 |
|  | Yield of rice (J/ha) | 1.01E+11 |  | *2.36E+05^e^* |  |

^a^ RNF and UEV reference for respective row numbers (converted to 12.0E+24 sej/year baseline): 1, 2, 3, Brown and Ulgiati (2016); 4, 5, Odum (1996); 6 Yang and Chen (2014); 7, 8, 9, 10, 13 Lan et al. (2002); 11, 12 Lou et al. (2015); 14, Wang et al. (2017).

^b^ The organic fertilizer contained 45.4% organic matter, 2.0% N, 2.9% P_2_O_5_, 1.2% K_2_O, and 29.1% water.

^c^ The raw data of 1.70E-01 year/ha corresponds to 15300 ¥/ha.

^d^ According to emergy theory by Brown and Ulgiati (1997), feedback yield energy are reused in the system, therefore, the emergy values are zero.

^e^ Italic: Calculated from this study.

Table E

Emergy analysis of rice monoculture mode for conventional rice production (/ha/yr).

| **NO.** | **Item** | **Raw data** | **RNF^a^** | **UEV^a^ (sej/ unit)** | **Emergy (sej)** |
| --- | --- | --- | --- | --- | --- |
|  | Free local renewable resources (L_R_) |  |  |  |  |
| **1** | Solar energy (J/ha) | 2.24E+13 | 1.00 | 1 | 2.24E+13 |
| **2** | Wind energy (J/ha) | 4.98E+09 | 1.00 | 8.00E+02 | 3.33E+12 |
| **3** | Rain chemical energy (J/ha) | 4.19E+10 | 1.00 | 7.00E+03 | 2.79E+14 |
| **4** | River water (Chemical) (J/ha) | 2.73E+10 | 1.00 | 6.50E+04 | 1.77E+15 |
|  | Free local nonrenewable resources (L_N_) |  |  |  |  |
| **5** | Energy of net soil loss (J/ha) | 3.41E+09 | 0.00 | 9.40E+04 | 3.21E+14 |
|  | Economic imported resources (F) |  |  |  |  |
| **6** | N fertilizer (g/ha) | 4.35E+05 | 0.00 | 4.84E+09 | 2.11E+15 |
| **7** | Compound fertilizer (g/ha) | 6.00E+05 | 0.00 | 3.56E+09 | 2.14E+15 |
| **7** | Pesticide (g/ha) | 8.40E+03 | 0.00 | 1.89E+10 | 1.59E+14 |
| **8** | Machine and tools g | 1.09E+04 | 0.00 | 8.57E+09 | 9.32E+13 |
| **9** | Electricity (J/ha) | 2.26E+08 | 0.09 | 2.04E+05 | 4.62E+13 |
| **10** | Diesel (g/ha) | 6.31E+04 | 0.00 | 4.09E+04 | 2.58E+09 |
| **11** | Labor^b^ (yr/ha) | 2.03E-02 | 0.60 | 4.98E+16 | 1.01E+15 |
| **12** | Services (¥/ha) | 9.56E+03 | 0.00 | 7.54E+11 | 7.21E+15 |
| **13** | Rice seeds (J/ha) | 9.12E+08 | 1.00 | 2.55E+05 | 2.33E+14 |
|  | Feedback yield energy in the system^c^ |  |  |  |  |
| **14** | Straw (J/ha) | 6.25E+10 | 0.37 | 5.99E+03 | 0.00 |
|  | Yield of rice (J/ha) | 1.47E+11 |  | *1.07E+05^d^* |  |

^a^ RNF and UEV reference for respective row numbers (converted to 12.0E+24 sej/year baseline): 1, 2, 3, Brown and Ulgiati (2016); 4, 5, Odum (1996); 6 Yang and Chen (2014); 7, 8, 9, 10, 13 Lan et al. (2002); 11, 12 Lou et al. (2015); 14, Wang et al. (2017).

^b^ The raw data of 2.03E-02 year/ha corresponds to 1824 ¥/ha.

^c^ According to emergy theory by Brown and Ulgiati (1997), feedback yield energy are reused in the system, therefore, the emergy values are zero.

^d^ Italic: Calculated from this study.

References

Brown, M.T., and Ulgiati, S. (1997). Emergy-based indices and ratios to evaluate sustainability: monitoring economies and technology toward environmentally sound innovation. *Ecol. Eng*. 9, 51-69. doi: 10.1016/S0925-8574(97)00033-5

Brown, M. T., and Ulgiati, S. (2016). Emergy assessment of global renewable sources. *Ecol. Model.* 339, 148–156. doi: 10.1016/j.ecolmodel.2016.03.010

Cavalett, O., Queiroz, J. F., and Ortega, E. (2006). Emergy assessment of integrated production systems of grains, pig and fish in small farms in the south Brazil. *Ecol. Model.* 193, 205-224. doi: 10.1016/j.ecolmodel.2005.07.023

Lan, S. F., Qin, P., and Lu, H. F. (2002). *Energy assessment of eco-ecological systems* (Beijing, China: Press, Chemical Industry).

Li, L., Lu, H., Ren, H., Kang, W., and Chen, F. (2011). Emergy evaluations of three aquaculture systems on wetlands surrounding the pearl river estuary, China. *Ecol. Indicat.* 11, 526-534. doi: 10.1016/j.ecolind.2010.07.008

Lou, B., Qiu, Y., and Ulgiati, S. (2015). Emergy-based indicators of regional environmental sustainability: a case study in shanwei, guangdong, China. *Ecol. Indicat.* 57, 514-524. doi: 10.1016/j.ecolind.2015.03.017

Odum, H. T. (1996). *Environmental accounting: Emergy and environmental decision making* (New York: Wiley).

Wang, X., Li, Z., Long, P., Yan, L., Gao, W., Chen, Y., et al. (2017). Sustainability evaluation of recycling in agricultural systems by emergy accounting. *Resour. Conserv. Recycl*. 117, 114-124. doi: 10.1016/j.resconrec.2016.11.009

Xi, Y. G., and Qin, P. (2020). Emergy evaluation of organic rice-duck mutualism system. *Ecol. Eng*. 35, 1677-1683. doi: 10.1016/j.ecoleng.2007.11.006

Yang, J., and Chen, B. (2014). Emergy analysis of a biogas-linked agricultural system in rural China-a case study in gongcheng yao autonomous county. *Appl. Eng*. 118, 173-182. doi: 10.1016/j.apenergy.2013.12.038

Yang, B. J., Sun, S., Chen, H. J., and Huang, G. Q. (2017). Research on emergy analysis and sustainability evaluation under paddy-upland rotation systems. *Ecol. Sci*. 36, 123-131. doi: 10.14108/j.cnki.1008-8873.2017.01.017
